# Microbial extracellular vesicles in the lung: friends in health, agents in disease

**DOI:** 10.20517/evcna.2025.139

**Published:** 2026-04-15

**Authors:** Marta Pagnini, Maria Conti, Giorgia Puddu, Matteo Daverio, Alessandro Celi, Erica Bazzan, Tommaso Neri

**Affiliations:** ^1^Dipartimento di Patologia Chirurgica, Medica, Molecolare e dell’Area Critica, University of Pisa, Pisa 56126, Italy.; ^2^Centro Dipartimentale di Biologia Cellulare Cardiorespiratoria, University of Pisa, Pisa 56126, Italy.; ^3^Department of Cardiac, Thoracic, Vascular Sciences and Public Health, University of Padova, Padova 35122, Italy.; ^4^Centro Cardiologico Monzino IRCCS, Milan 20138, Italy.; ^#^These authors contributed equally to this work as first authors.; ^§^These authors contributed equally to this work as senior authors.

**Keywords:** Microbial extracellular vesicles, respiratory diseases, probiotic extracellular vesicles, host-microbe interactions, postbiotics

## Abstract

Microbial extracellular vesicles (mEVs) are emerging as key mediators at the host-microbe interface in the lung, playing a remarkable dual role as both pathogenic drivers and therapeutic modulators. These nano-sized, membrane-bound structures (20-400 nm) secreted by bacteria, fungi, and other microorganisms carry diverse bioactive cargo including lipids, proteins, and nucleic acids that can profoundly influence host immune responses and tissue homeostasis. mEVs derived from probiotic bacteria demonstrate significant therapeutic potential as immunomodulatory agents capable of reducing pulmonary inflammation and enhancing epithelial barrier function. These probiotic-derived vesicles represent a novel class of postbiotics - bioactive microbial products that confer health benefits without requiring live microbial cells. Conversely, in pathogenic contexts, mEVs from bacteria such as *Pseudomonas aeruginosa* and *Escherichia coli* trigger robust inflammatory responses in the lung through pattern recognition receptor activation, particularly Toll-like receptors (TLRs)-2 and TLR-4, leading to upregulation of pro-inflammatory cytokines and contributing to chronic respiratory conditions including asthma and chronic obstructive pulmonary disease. Given its extensive surface area and highly specialized immune network, the lung represents a highly receptive site for mEV-mediated interactions. This review synthesizes evidence on pathogenic mechanisms of mEVs and explores their therapeutic potential in respiratory medicine, with specific focus on: (1) the role of environmentally-derived mEVs from dust and airborne sources in chronic respiratory inflammation; (2) recent experimental evidence of probiotic-derived mEVs therapeutic effects across diverse pulmonary conditions and (3) the concept of mEVs as both protective postbiotics and inflammatory triggers in the lung.

## INTRODUCTION

The current review provides a comprehensive up-to-date overview of the dual role of mEVs in pulmonary health and disease, filling an important gap in our knowledge of these multifaceted host-microbe interactions. This review examines the emerging therapeutic potential of probiotic-derived extracellular vesicles (EVs) as immunomodulatory agents in pulmonary inflammation. It also explores their role as postbiotic bioactive microbial products capable of conferring health benefits independently of live microorganisms. Finally, it synthesizes current evidence on the pathogenic mechanisms through which microbial EVs contribute to respiratory diseases, including chronic infection, asthma, and chronic obstructive pulmonary disease (COPD). Additionally, current limitations in experimental models and clinical translation are discussed, and key future research directions necessary to advance mEV-based therapeutic strategies in respiratory medicine are outlined. By integrating these perspectives, we wish to establish a conceptual framework accepting mEVs as not only pathogenic mediators but also protective modulators in the lung, ultimately ending in novel therapeutic strategies in respiratory health management.

## MICROBIAL EXTRACELLULAR VESICLES: CHARACTERISTICS AND FUNCTIONAL ROLES

### Microbial EVs: definition, origin and composition

**Figure 1:**
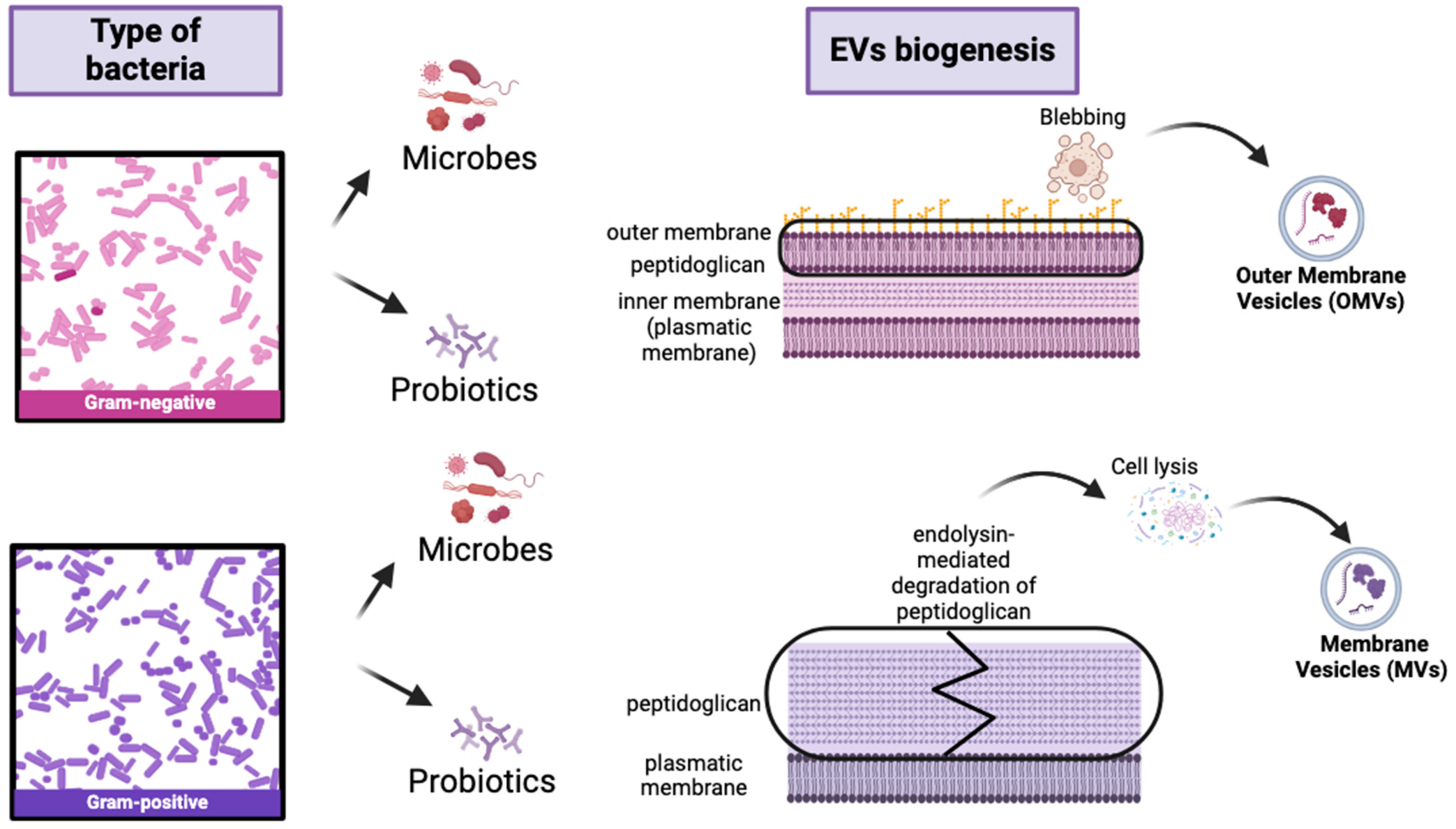
Schematic representation of generation of extracellular vesicles from Gram-negative and Gram-positive bacteria. OMVs generate from Gram-negative bacteria through outer membrane blebbing; instead, MVs generate from Gram-positive bacteria through cell membrane budding and cell wall remodelling caused by degradation of peptidoglycan resulting in cell lysis. Created in BioRender. Mengozzi, A. (2026) https://BioRender.com/o84qsps. EVs: Extracellular vesicles; OMVs: outer membrane vesicles; MVs: membrane vesicles.

**Figure 2:**
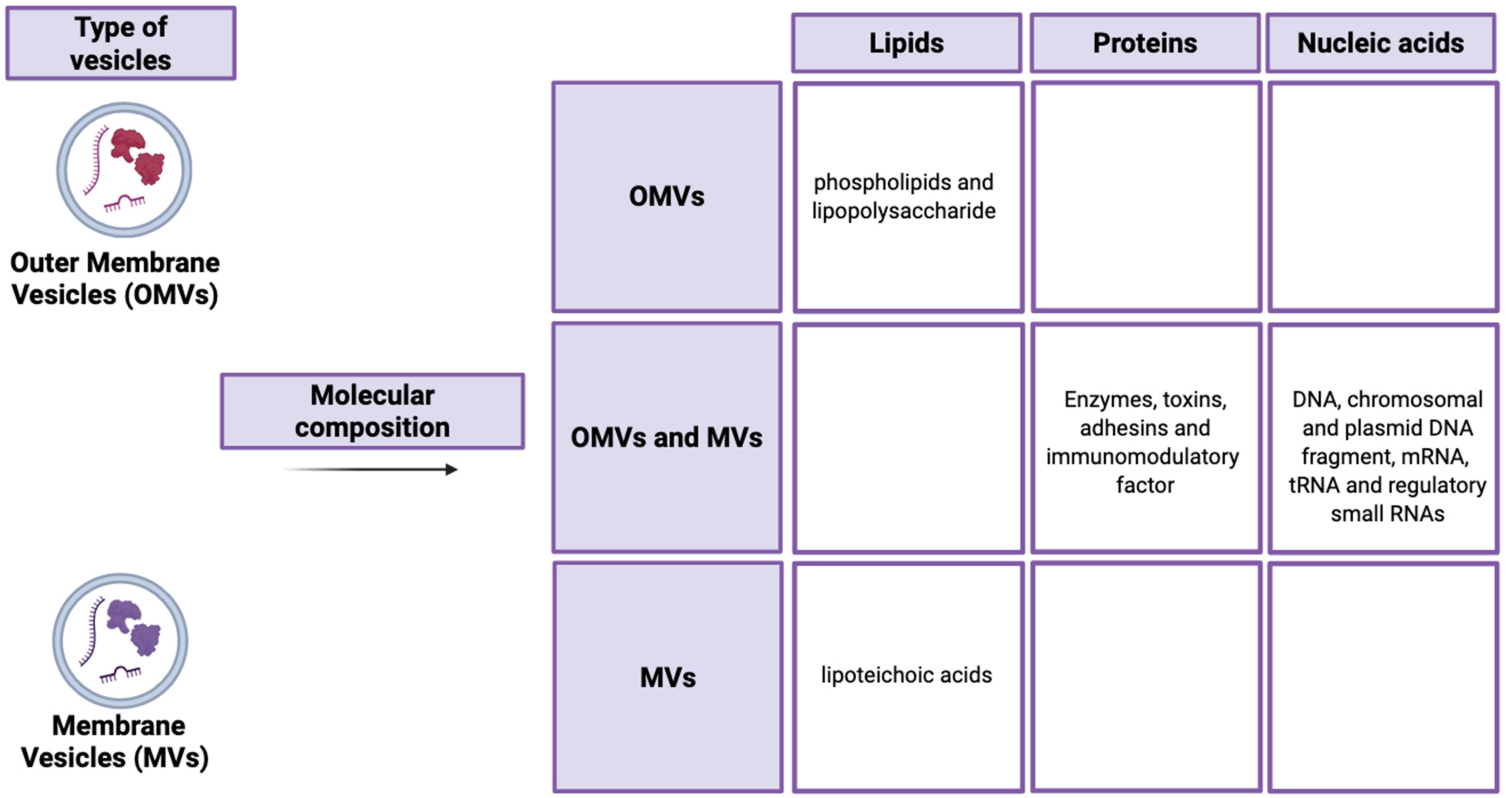
Schematic representation of molecular composition of extracellular vesicles, highlighting the similarities and differences between OMVs and MVs in term of their lipid, protein and nucleic acid content. Created in BioRender. Mengozzi, A. (2026) https://BioRender.com/2zq9ihg. OMVs: Outer membrane vesicles; MVs: membrane vesicles.

Microbial extracellular vesicles (mEVs), by definition, are nano-sized, membrane bound extracellular structures, generally in the 20-400 nm range, that are secreted from microorganisms into their external environment. mEVs carry a wide variety of bioactive cargo and are important components of intracellular communication and host-microbe interaction^[1]^. mEVs production is a biological mechanism that is observed as an effect of all domains of life, including bacteria, archaea, fungi and parasites^[2]^. The classification and nomenclature of mEVs differ on their origin. Gram-negative bacteria mainly secrete outer membrane vesicles (OMVs), while Gram-positive bacteria release membrane vesicles (MVs) through different biogenesis pathways^[3,4]^. In Gram-negative bacteria, OMV generation is often driven by the accumulation of envelope proteins or peptidoglycan fragments that generate turgor pressure, causing the outer membrane to bulge and form vesicles. Alternatively, the downregulation or modification of cross-linking proteins, including Braun’s lipoprotein (Lpp), can reduce membrane-cell wall coupling and promote vesicle release^[5]^. In Gram-positive bacteria, which possess a thick peptidoglycan cell wall, vesicle release is more complex. It often requires the local activity of endolysins, which degrade peptidoglycan to create pores through which the cytoplasmic membrane can protrude and release vesicles^[6]^. These mechanisms highlight that mEV biogenesis is species- and context-dependent, providing a foundation for understanding their diverse composition and functional roles in host-microbe interactions. In Gram-negative bacteria, for example, EVs originate from outer membrane blebbing, producing OMVs that enclosed periplasmic components within a lipid bilayer composed of Lipopolysaccharide (LPS), phospholipids and membrane protein^[7]^. In contrast, Gram-positive bacteria lack an outer membrane but produce MVs through processes involving cell wall remodeling enzymes. These enzymes create transient openings in the thick peptidoglycan layer, allowing the cytoplasmic membrane to extrude and release vesicles [Figure 1]^[8]^. Probiotic bacteria, such as *Lactobacillus* and *Bifidobacterium*, generate EVs with varied compositions, which can underlie their health-promoting effects. Recent studies have shown that probiotic-derived EVs contain strain-specific proteins, lipids and genetic material that can modulate host immune response and maintain gut homeostasis^[9,10,11]^. The composition of mEVs is extremely heterogeneous, including membrane-forming lipids [phospholipids and LPS in Gram-negative bacteria; lipoteichoic acid (LTA) in Gram-positive bacteria], membrane and cytosolic proteins such as enzymes, toxins, adhesins, and immunomodulatory factors, as well as nucleic acids (i.e. chromosomal and plasmid DNA fragments, mRNA, tRNA, and small regulatory RNAs) [Figure 2]^[5,12]^. This complex cargo is not randomly packaged but often selectively sorted, suggesting sophisticated mechanisms that determine EVs content based on environmental conditions and bacterial physiological states^[13]^. The lipid composition of EVs largely reflect the membrane of origin but is often enriched in specific lipids that contribute to membrane curvature and vesicle stability, with phosphatidylglycerol and cardiolipin being particularly prevalent in bacterial EVs^[14,15]^.

The diversity of bacteria, bacterial EVs and source material characteristics make it difficult to establish universal recommendations for sample type, pre-processing, separation, collection and characterisation. Despite this heterogeneity, some recommendations are outlined in the latest Minimal Information for Studies of Extracellular Vesicles (MISEV) guidelines^[16]^ edited by the International Society for Extracellular Vesicles (ISEV), developed to improve the rigor, reproducibility, and comparability of research involving EVs.

Most of the considerations established for the analysis of eukaryotic-derived EVs are also applicable to bacterial EVs and mainly involve three interconnected aspects: culture conditions, methodological details of separation and concentration, and characterisation of EV preparations.

To ensure research rigor and inter-laboratory comparability, strict adherence to standardized reporting guidelines, particularly the MISEV adapted to the microbial context, is essential.

With regard to culture conditions, especially for culture-derived bacterial EVs, key parameters such as oxygenation/aeration, culture format (e.g., standing or shaking), and growth phase at harvest should be systematically reported, as they can significantly influence EV release, composition, and downstream functional interpretation. Methodological aspects of separation and concentration represent one of the major standardization challenges in bacterial EV research. Non-specific isolation methods, including precipitation and differential ultracentrifugation, may co-isolate substantial amounts of non-EV materials such as pili, flagella, bacteriophages, and protein-, Lpp-, or nucleoprotein complexes. This co-isolation constitutes a critical confounding factor for functional studies.

In line with MISEV recommendations, the use of orthogonal purification approaches - such as size exclusion chromatography (SEC) or density gradient ultracentrifugation - together with transparent reporting of protein and non-EV contamination levels, is strongly encouraged.

Finally, the characterisation of bacterial EV preparations should rely on the combined application of at least three orthogonal approaches: (i) morphological analysis by transmission or scanning electron microscopy (TEM/SEM); (ii) particle size and concentration measurements using techniques such as nanoparticle tracking analysis (NTA) or tunable resistive pulse sensing (TRPS); and (iii) biochemical assessment of composition and purity. LPS and LTA remain broadly used markers for Gram-negative and Gram-positive bacteria, respectively, supported by well-characterised and commercially available affinity reagents. However, for many bacterial species, specific markers capable of reliably discriminating EVs from non-EV contaminants are still lacking, further underscoring the need for multi-parameter characterisation strategies^[16]^.

### Microbial EVs: biological function and interaction with host cells

mEVs are complex communication tools between microorganisms and host cells, as efficient multipurpose mediators with the ability to regulate numerous biological processes among and within kingdoms^[17]^. The biological functions of mEVs are highly diverse, including bacterial communication, biofilm formation, pathogenesis, and modulation of the host immune response. In pathogenic settings, mEVs are able to carry virulence factors, toxins, and immunomodulatory molecules to host cells directly, thus facilitating bacterial colonization and evading host immune surveillance^[18]^. Conversely, commensal and probiotic bacteria-derived EVs are likely to possess protective and anti-inflammatory roles which contribute to intestinal homeostasis and barrier integrity^[19,20]^. The host cell-mEV interaction involves several different entry mechanisms which enable these nanostructures to transfer their bioactive cargo effectively. Endocytosis is a primary uptake pathway, with multiple subtypes operating in different host cell types. These include clathrin-mediated endocytosis, caveolin-dependent endocytosis, macropinocytosis, and lipid raft-mediated internalization. Direct membrane fusion is another significant entry mechanism where mEV lipids merge with host cell plasma membrane, enabling the direct import of vesicular contents into the cytoplasm without endosomal processing^[21]^. In addition, receptor-mediated recognition plays a vital role in mEV-host cell interaction as well, where Pattern Recognition Receptors (PRRs) such as Toll-like receptors (TLRs) and nucleotide-binding oligomerization domain (NOD)-like receptors identify bacterial components in EVs and trigger some downstream signalling processes^[22]^. Looking closer at the molecular “handshake” between mEVs and the lung, it is clear that surface-level recognition is just the starting point. For many Gram-negative pathogens, the Outer Membrane Protein A (OmpA) on the vesicle surface acts as a critical anchor, binding to host receptors and facilitating TLR engagement and subsequent Myeloid differentiation primary response gene 88 (MyD 88)- or TIR-domain-containing adapter-inducing interferon-beta (TRIF)-dependent signaling to kickstart the TLR response^[23]^. But the real damage often happens after the vesicle is inside. Once mEVs are taken up - often via endocytosis - their cargo of peptidoglycans can leak into the host cytosol, where they are “sensed” by nucleotide-binding oligomerization domain-containing protein 1 (NOD1)/NOD2 receptors^[24]^. This isn’t just a minor signal; it often contributes to NLRP3 inflammasome priming and activation^[25]^. This specific molecular complex activates Caspase-1, which is the “trigger” for releasing mature Interleukin (IL)-1β and IL-18 - two heavy-hitters in pulmonary inflammation. Furthermore, recent evidence suggests that mEV-derived lipids aren’t just structural; they can actively disrupt host mitochondria^[26]^, causing a spike in mitochondrial reactive oxygen species (mtROS) that sustains Nuclear Factor-kappa B (NF-κB) activation and delays inflammatory resolution^[27]^. Through these complex mechanisms, mEVs exert a powerful influence over the host’s immune landscape. During host cell interaction, mEVs also influence immune responses strongly through complex mechanisms. They can initiate pro-inflammatory cascades through the recognition of pathogen-associated molecular patterns (PAMPs) by host PRRs, leading to the cytokine and chemokine production that coordinates antimicrobial immune responses^[28]^. Furthermore, certain mEVs, also have the ability to suppress immune responses by transferring immunomodulatory proteins that interfere with NF-κB signaling or other inflammatory pathways^[29]^. Along with immune modulation, mEVs play an important role in the physiology of epithelia as well, influencing barrier function, proliferation, and differentiation^[30]^. In the gut milieu, EVs of probiotic and commensal bacteria contribute to epithelial barrier function by inducing the expression of tight junction proteins, increasing the physical barrier against pathogen entry^[31]^. In addition, mEVs can carry small RNAs that control host gene expression epigenetically control and have the potential to influence long-term cellular processes and adaptive responses^[32]^.

### The lung as a privileged target for microbial EVs

The lung is a privileged mEVs’ target due to its unique anatomical, physiological, and immunological characteristics that enable persistent contact with environmental microorganisms and their secreted products^[33]^. With an extensive surface area of approximately 70-100 m^2^ and exposure to 10,000-15,000 litres of inhaled air daily, the pulmonary system represents a major host-environment interface, continuously encountering airborne microorganisms and particulate matter^[34]^. This high environmental exposure has driven the evolution of sophisticated immune defenses, positioning the lung as an immunologically hypervigilant organ. It harbors a complex network of resident immune cells, including alveolar macrophages, dendritic cells, and lymphocytes, that collectively maintain a delicate balance between tolerance and inflammation^[35]^. Adding to this complexity is the presence of a distinct pulmonary microbiota. Contrary to the historical perception of lung as sterile environment, recent advances in microbiome research have revealed the presence of a diverse resident microbiota with approximately 10^3^-10^5^ bacterial cells per gram of tissue, predominantly belonging to the *Firmicutes, Bacteroidetes, and Proteobacteria* phyla^[36]^. Although lower in biomass compared to other mucosal sites, this microbiota plays a critical role in shaping local immunity and maintaining tissue homeostasis^[37]^. Its composition is dynamically regulated through microaspiration from the oropharyngeal cavity, mucociliary clearance, and immune surveillance^[38]^. In this complex pulmonary environment, mEVs play crucial roles in modulating immune response. EVs from commensal bacteria can induce immunological tolerance by stimulating the regulatory T cells and promoting anti-inflammatory cytokines, suppressing the overactive inflammatory responses against non-harmful environmental antigens^[39]^. Importantly, mEVs can cross the pulmonary epithelial barrier and modulate immune responses without direct bacterial contact.

### The gut-lung axis: its role in mEV-mediated immunity

Evidence from experimental and clinical trials indicates that the gastrointestinal tract and the lungs participate in a constant bidirectional interaction known as the gut-lung axis; although these organs are anatomically distinct and functionally specialized, research suggests that they share immune, neural and microbial connections that allow them to influence each other’s physiological state, including metabolic and immunological dimensions^[40,41,42]^.

Among the mediators of inter-organ communication, EVs secreted by host and microbial cells have emerged as key players, carrying bioactive cargos that regulate immune modulation and tumor progression^[43]^.

They are, in fact, integral to numerous physiological and pathological processes, owing to their ability to transport physiologically active macromolecules, nucleic acids and immune modulators. These cargos can significantly modify the biological characteristics of targeted cells, influence epithelial permeability, T-cell priming and cytokine networks, thereby affecting respiratory immunity and systemic health^[43,41]^.

Their high stability and favorable safety profile enhance their potential as complementary or preventive therapeutic options across a range of clinical applications^[41]^.

In particular, growing evidence indicates that bacterial EVs are key effectors of the gut-lung axis. Gut-derived EVs can cross the intestinal barrier, enter systemic circulation, and deliver microbial proteins, LPS, and nucleic acids to distant organs including the lungs^[43]^. In addition, EVs can transport microbial metabolites originating from the gut, like short-chain fatty acids (SCFAs) and secondary bile acids (BAs), modulating immune cell behaviour and inflammation within the lungs. Furthermore, compromised intestinal barrier integrity with heightened permeability may facilitate the translocation of microbial byproducts and pro-inflammatory mediators from the gut lumen into the systemic circulation, thereby exacerbating pulmonary fibrotic processes^[40]^.

Within the pulmonary microenvironment, these vesicles interact with alveolar macrophages and dendritic cells, and modulate cytokine release via TLR signaling, as well as both inflammatory and antitumor responses. This vesicle-mediated communication complements established mediators such as SCFAs and provides a mechanistic rationale for how the gut microbiota exert direct influence on lung immunity and potentially induce lung carcinogenesis^[43]^.

## EVs FROM PROBIOTICS: MODULATORS OF THE RESPIRATORY IMMUNE RESPONSE

### Probiotics-derived EVs: their production from non-pathogenic bacteria and their advantages

Probiotics are defined as “live microorganisms which, when administered in adequate amounts confer a health benefit on the host” [FAO (Food and Agriculture Organization) and WHO (World Health Organization), 2001]^[44]^. These beneficial microorganisms play multiple roles in the human body and exert their effects through various mechanisms, including immune modulation, prevention of pathogenic bacteria colonization (barrier effect), and the improvement of mucosal barrier integrity and function^[45]^. Like pathogenic bacteria, probiotics can produce EVs^[46]^. While the role of EVs in the virulence of life-threatening pathogens is well established and extensively described in the literature, considerably less attention has been devoted to the health-promoting effect of EVs secreted by non-pathogenic microorganisms, including probiotics. However, in recent years, awareness of probiotic-EVs as a promising therapeutic platform has grown rapidly^[47]^. Probiotics-EVs possess several unique advantages that make them particularly attractive for therapeutic applications. Their nanometric size allows them to easily diffuse through various biological environments and target cells located far from the parent bacteria^[30]^. Furthermore, these vesicles demonstrate remarkable stability and can be produced in large quantities under controlled conditions^[48]^. Unlike whole probiotics cells, probiotics-EVs can effectively penetrate barriers such as the gastrointestinal and blood-brain barriers, spread across the mucus layer and directly migrate to distant tissues^[49]^. Due to their ability to carry many different bioactive macromolecules, including virulence factors, nucleic acids, lipids and various bioactive compounds, that can effectively modulate the biological properties of target cells, probiotic-EVs have recently been proposed as an independent type of secretion system, called type-0^[30,50]^.

### Probiotics-derived EVs as postbiotics

Since probiotic-EVs are secretory components directly associated with the health benefits of probiotic bacteria, they can be considered postbiotics^[50]^. Postbiotics are defined as “a preparation of inanimate microorganisms and/or their components that confers a health benefit on the host.” This classification has gained particular relevance given the safety concerns associated with the use of live bacterial strains, especially in children and vulnerable patients, primarily due to the risk of bacterial translocation from the intestine to the systemic circulation^[51]^. Following bacterial inactivation, typically through heat treatment, dead cells can release bacterial components that retain key immunomodulatory effects and antagonistic activity against pathogens. Critical components, such as LTA, peptidoglycans and exopolysaccharides (EPS), have been proposed to be mainly involved in health-promoting properties. These include maintenance of proper resident microbiota structure, strengthening of the host epithelial barrier, modulation of local and systemic immune response, and enhancement of the host metabolic activity^[51,47,50]^. Importantly, these bioactive components can be incorporated into EVs and transported throughout the body, potentially enhancing their stability and enabling targeted delivery to specific tissues.

### Functional properties of probiotics-derived EVs: focus on anti-inflammatory effects and reinforcement of epithelial barrier

Extensive research has been conducted using both *in vitro* and *in vivo* models to elucidate the diverse functions of probiotic-EVs^[47]^. Communication between commensal bacteria and the host via EVs can result in varied outcomes, ranging from pro-inflammatory and cytotoxic effects to non-immunogenic and beneficial responses. These outcomes depend on several factors, including the bacteria species, the type of target cells, and the quantity of vesicles internalized^[49]^. EVs derived from probiotics have demonstrated significant potential as immunomodulatory agents due to their anti-inflammatory effects and ability to influence host immune responses. These vesicles interact with immune cells through their bioactive molecules, thereby influencing the production of cytokine, chemokine and antibody production^[47,48]^. In particular, probiotic-EVs can reduce the expression of systemic inflammatory biomarkers including IL-6, IL-8 and TNF-α, partially through modulation of the NF-κB pathway, a crucial regulator of inflammation. Additionally, they demonstrate the ability to stimulate the innate immune system, enhance antigen processing, and undergo clathrin-mediated endocytosis, highlighting their potential as adjuvants in immunotherapy^[48]^.

There is a growing body of evidence supporting the role of probiotic bacteria and their secreted products, particularly EVs, in promoting epithelial barrier integrity. While understanding the efficacy of individual species and the mechanisms by which microbial-derived products promote barrier functions remain critical, multiple candidate mechanisms have been identified. These include pH modification through lactic acid or short-chain fatty acid production, lipid-derived induction of anti-inflammatory innate and adaptive immune responses, and activation of toll-like receptor signaling pathways, which may act synergistically to promote epithelial integrity^[52]^. The principles of epithelial barrier protection extend beyond the gastrointestinal tract to the respiratory system, where epithelial cell layers lining the airway and alveoli form critical defensive barriers against pathogenic microorganisms’ invasion. These respiratory epithelial barriers serve as essential protective structures for lung homeostasis. Within these tissues, tight junction proteins regulate diverse intracellular signaling pathways and are fundamental for controlling material exchange between lung cell environments. During inflammatory conditions, the release of numerous proinflammatory mediators triggers a cascade of negative events, including cell apoptosis, pulmonary edema, alveolar gas exchange dysfunction and compromise of lung epithelial barrier integrity. This inflammatory cascade results in increased epithelial permeability to water and proteins, creating conditions that predispose to secondary infection and can ultimately progress to acute lung injury (ALI)^[53]^. Probiotics-EVs demonstrate therapeutic potential in the respiratory context by enhancing lung epithelial barrier function through several mechanisms. They can regulate the expression of critical tight junction proteins, including occludin and ZO-1 (zonula occludens-1), thereby stabilising the distribution and function of lung barrier proteins. Additionally, probiotic-EVs reduce apoptosis of lung tissue cells, contributing to the preservation of epithelial barrier integrity and respiratory function^[53]^.

To conclude, the effects of probiotic-derived EVs are highly strain-dependent: differences in protein, lipid and RNA cargo between strains can produce divergent immunomodulatory outcomes. Therefore, candidate therapeutic EVs require detailed molecular profiling and functional assays to establish reproducible activity and safety.

### Experimental evidence of beneficial effects of probiotic-derived EVs on pulmonary inflammation: *in vivo* and *in vitro* studies

The previous sections provided a general overview of the characteristics and functions of probiotic-derived EVs, as well as the mechanistic pathways through which they modulate inflammatory responses and epithelial barrier integrity. The focus now shifts to experimental evidence supporting their functional relevance in pulmonary disease models, with particular attention to findings from *in vivo* and *in vitro* studies.

Within this mechanistic framework, increasing attention has been directed toward the investigation of probiotic-derived EVs at the pulmonary level, and numerous studies have demonstrated their beneficial effects across various lung diseases.

Among these conditions, ALI represents a well-established experimental model that closely recapitulates key inflammatory and barrier-disruptive processes described in the previous sections, making it particularly suitable for evaluating the therapeutic potential of probiotic-derived EVs.

ALI represents a critical pulmonary condition caused by multiple factors including infection, trauma, shock and aspiration. ALI is characterised by alveolar epithelial barrier damage, inflammatory cells accumulation, pulmonary edema, and increased vascular permeability^[53]^. Given its high mortality rates, identifying an effective treatment remains a clinical priority. Liu *et al*., conducted an *in vivo* study using LPS-induced ALI in mice to investigate the therapeutic potential of probiotic-EVs. They demonstrated that pretreatment with either *Clostridium butyricum* or its EVs significantly reduced the concentration of pro-inflammatory cytokines in serum, bronchoalveolar lavage fluid (BALF), and lung tissues, compared to the LPS control group. In addition, nasal administration of these EVs enhanced the expression of tight junction proteins ZO-1 and occludin in the lung tissue, supporting epithelial barrier restoration^[53]^. Complementing these findings, Zhao *et al*., investigated the relationship between ferroptosis and ALI progression, focusing on macrophage function. Macrophages play a crucial role in pulmonary immune responses and are intricately involved in ALI development. Ferroptosis influences macrophage polarization through iron metabolism modulation and metabolic reprogramming, ultimately affecting inflammatory factor production, tissue repair, and immune regulation^[54]^. In ALI, there is a significant upregulation of intercellular Fe2+ content and reactive oxygen species (ROS) accumulation in both *in vivo* and *in vitro* macrophage studies. This study demonstrated that EVs derived from *Lactiplantibacillus plantarum* effectively counteract these pathological alterations, providing a protective effect against LPS-induced ALI through their ability to inhibit macrophage polarization and attenuate ferroptosis.

These findings propose probiotic-derived EVs as a promising therapeutic approach for ALI prevention and treatment, highlighting the potential of developing therapeutic such strategies based on probiotic-derived biomolecules^[54]^.

Beyond acute inflammatory conditions such as ALI, probiotic-derived EVs have also been investigated in chronic inflammatory airway diseases, including asthma.

Asthma represents a heterogeneous and multifactorial disease characterized by chronic airways inflammation, reversible airflow limitation and bronchial hyperreactivity, involving complex interactions between genetic and environmental factors^[55,56]^. This disorder can be associated with T helper type 2 (Th2) cell-dependent immune responses, eosinophilia and IgE production. During pathogenesis, Th2 cells produce various cytokines, including IL-5, IL-9 and IL-13^[57]^. Neutrophils also play a pivotal role, as their massive infiltration in the airways induces frequent asthma exacerbations and steroid resistance^[56]^. Clinical studies in children with asthma have demonstrated that probiotics, administered alone or in combination, can reduce the number of exacerbations, improve lung function, alleviate respiratory symptoms and attenuate lung inflammation^[55,58,59,60]^. Although the precise mechanism by which EVs are involved in asthma pathogenesis remains unclear, they represent potential therapeutic agents for asthma treatment. Lee and colleagues proposed probiotic-derived EVs as key molecules with functional effects on immune regulation. They have demonstrated that in allergic asthmatic mice, EVs from *Lactococcus lactis* decrease IL-5 and IL-13 levels while increasing interferon gamma (IFN-γ) in the BALF, suggesting a shift from Th2 to Th1 immune response^[57]^. Another comprehensive study examined extracellular vesicles from *Micrococcus luteus* (MlEVs) using asthmatic patients, mouse models and A549 (human alveolar epithelial) cells. In asthmatic patients, decreased levels of MlEVs-specific immunoglobulin G subclass 4 (IgG4) showed clinical significance and correlated with reduced lung function compared to healthy controls. When LPS-induced asthmatic mice received intranasal MlEVs treatment, the EVs significantly reduced neutrophil numbers, but not eosinophils, decreased IL-1β/IL-17 production in BALF, reduced immune cell infiltration and diminished epithelial thickness. The study also investigated airway epithelial cells, in particular A549, treated with or without MlEVs in the presence of LPS. The authors demonstrated that these EVs significantly reduced IL-8 production and p65 phosphorylation, both of which contribute to the secretion of chemoattractants for neutrophils^[56]^.

In line with these observations, probiotic-derived EVs have also been explored in the broader context of allergic airway diseases.

Allergic diseases represent some of the most common chronic inflammatory conditions, with allergen-specific immunotherapy being the only available disease-modifying treatment. Despite its high effectiveness, this approach has several disadvantages, including the need for long term administration and the occurrence of adverse reactions. Both genetic and environmental factors contribute to allergic disease, and the increasing prevalence has been linked to changes in microbial load due to lifestyle and hygiene modification. Studies have demonstrated that certain probiotics can reduce the inflammatory events, preventing allergic asthma and allergen-specific immune responses in mice. Schmid *et al*., investigated the effects of OMVs derived from *Escherichia coli* on immune activation and allergic airway inflammation using both *in vitro* models (splenocytes and bone marrow-derived dendritic cells) and *in vivo* models (mice with ovalbumin-induced allergic airway inflammation)^[61]^. The experiments showed that these EVs activated key innate immune receptors and stimulated both pro- and anti-inflammatory cytokine production in cells. Instead, intranasal administration of OMVs in the mouse model significantly reduced airway hyperresponsiveness, eosinophilia, Th2 cytokine levels, and mucus production. These positive effects highlight the potential of OMVs as safe and effective postbiotic therapy for allergic diseases^[61]^.

Beyond inflammatory and allergic airway disorders, emerging evidence suggests that probiotic-derived EVs may also influence tumor-related immune responses in the lung.

Lung cancer remains the leading cause of cancer-related deaths worldwide. While immunotherapy with agents such as anti-programmed death-1 and its ligand 1 (PD-1/L1) represents standard treatment for advanced non-small cell lung cancer (NSCLC), many patients do not derive direct benefit. Recent studies have investigated new strategies to improve antitumor response through gut microbiota regulation. One significant study demonstrated that commensal *Bifidobacterium*-derived EVs can modulate the therapeutic effect of anti-PD-1 therapy in NSCLC. These EVs can easily penetrate epithelial cells and modulate the PD-L1 expression, in both human (A549, H460) and murine (LL/2) lung cancer cells, as well as in non-cancerous immortalized lung epithelial cells (BEAS-2). In addition, using a syngeneic mouse model, the study showed that *Bifidobacterium*-derived EVs can synergize anti-tumor effect of anti-PD-1 therapy through modulation of key cytokines (upregulation of IFN-γ, IL-2), immune response and oncogenic pathways, and an increase in tumor-infiltrating CD8+ T-cells^[62]^. In addition to cancer-related applications, probiotic-derived EVs have been investigated in non-neoplastic chronic lung diseases characterized by aberrant inflammatory and fibrotic responses.

**Figure 3:**
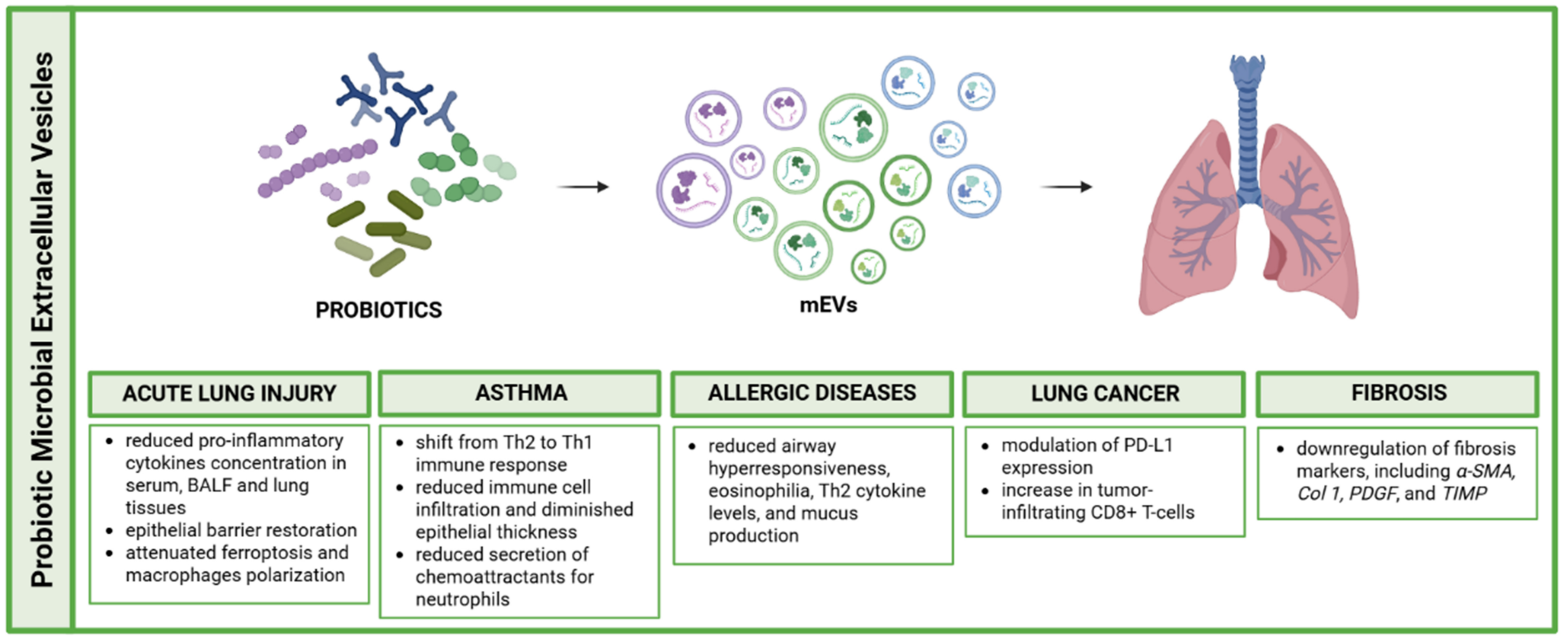
Schematic representation of probiotic microbial-derived extracellular vesicles roles in pulmonary disease modulation. Created in BioRender. Bazzan, E. (2025) https://BioRender.com/s6l04uq. mEVs: Microbial extracellular vesicles; BALF: bronchoalveolar lavage fluid; Th1: T helper 1; Th2: T helper 2; PD-L1: programmed death-ligand 1; CD8+: cluster of differentiation 8 positive T cells; α-SMA: alpha-smooth muscle actin; Col 1: collagen type I; PDGF: platelet-derived growth factor; TIMP: tissue inhibitor of metalloproteinases.

Pulmonary fibrosis represents a significant challenge in lung disease treatment, characterized by lung parenchyma scarring and compromised respiratory function with fatal outcomes and multifactorial etiology. The lack of effective non-invasive treatments underscores the need for targeted therapies to improve patient outcomes. Aziziraftar and colleagues conducted a study on mice with carbon tetrachloride (CCl4)-induced pulmonary fibrosis exacerbated by a high-fat diet (HFD). They demonstrated that administration of live and pasteurized *Akkermansia muciniphila*, as well as its EVs, exhibited significant antifibrotic effects in lung tissue. In particular, there was a downregulation of fibrosis markers, including *α-SMA, Col 1, PDGF*, and *TIMP*. In addition, both live and pasteurized bacteria, as well as their EVs, prevented overexpression of TNF-α, IL-1β and TGF-β in HFD mice, while elevating production of the anti-inflammatory cytokine IL-10^[40]^. Given the numerous *in vitro* and *in vivo* findings demonstrating the potential effects of probiotic-derived EVs at the pulmonary level, these vesicles may represent a valuable alternative or adjunct to conventional treatments used in various pulmonary diseases. The diverse mechanisms of action observed across different respiratory conditions (schematically summarized in Figure 3) suggest that probiotics-EVs could serve as versatile therapeutic tools in respiratory medicine.

## PATHOGENIC ASPECTS OF MEVS IN CHRONIC PULMONARY DISEASES

In addition to the beneficial effects exerted by probiotic-derived EVs, it is important to consider that bacterial EVs can also play a detrimental role. Pathogenic bacterial EVs can trigger robust inflammatory responses. Several studies have demonstrated that OMVs from pathogenic bacteria such as *Pseudomonas aeruginosa* and *Escherichia coli* provoke strong immune responses in the lung by activating PRRs, particularly TLR on airway epithelial cells and alveolar macrophages. Activation of TLR-2 and TLR-4 by OMV-associated components such as LPS results in the upregulation of proinflammatory cytokines including IL-6, TNF-α and IL-8. mEVs, particularly OMVs produced by Gram-negative bacteria, have emerged as key modulators in the pathogenesis of chronic respiratory diseases^[63,64]^. In the context of asthma, mEVs act as both microbial effectors and environmental inducers of inflammation. OMVs derived from respiratory pathogens or indoor dust can trigger either Th2- or Th17-skewed responses, leading to eosinophilic or neutrophilic airway inflammation depending on the disease endotype^[65,66,67]^. Beyond indoor dust, recent research has highlighted the broader environment as a critical reservoir of mEVs with distinct biological potential. A pivotal study by Yun *et al*. demonstrated that the composition of bacterial EVs differs significantly between air and soil environments^[68]^. Notably, while soil-derived bacterial EVs were enriched in metabolic pathways that suggest a potential impact on gut microbiota via ingestion, airborne bacterial EVs, a major component of inhaled dust, showed a higher abundance of genes related to bacterial pathogenicity, such as ATP-binding cassette trasporters (ABC) transporters and two-component systems. These findings suggest that inhaled environmental mEVs are not merely inert debris but bioactive particles capable of triggering specific immune responses in the respiratory tract, potentially distinct from those elicited by soil-associated vesicles.

Experimental evidence shows that OMVs can suppress major histocompatibility complex (MHC) expression on epithelial cells, disrupt tight junctions, and regulate host microRNA networks, contributing to epithelial dysfunction and persistent inflammation^[66,65,67]^. Clinical studies have identified differences in OMV composition among asthma phenotypes: for instance, non-eosinophilic asthma is associated with a higher abundance of OMVs from *Klebsiella* and a depletion of beneficial genera such as *Lactobacillus*^[66,67,69,70]^.

Additionally, asthmatic children frequently exhibit serum IgG responses against bacterial OMVs isolated from indoor dust, with sensitization rates exceeding 50%, compared to less than 5% in children affected by other allergic diseases, as reported by Kim *et al*.^[67]^. These findings suggest that early-life exposure to bacterial EVs, whether endogenously produced by the resident microbiota or environmentally acquired, may contribute to asthma development by shaping host immune responses. Beyond their potential role in disease pathogenesis, OMV-specific antibody responses highlight the emerging relevance of microbial EVs as non-invasive biomarkers. Owing to their intrinsic stability and detectability in accessible biofluids, including serum and BALF, microbial EVs and anti-OMV antibodies are increasingly being considered within the framework of liquid biopsy approaches for chronic lung diseases. Nevertheless, the clinical translation of OMV-based biomarkers remains limited by challenges related to specificity, microbial heterogeneity, and standardization, underscoring the need for further validation in larger, well-characterized cohorts^[67]^. Similar mechanisms are observed in COPD, which represents one of the most common chronic respiratory disorders, mainly caused by long-term exposure to noxious particles or gases, with cigarette smoke being the principal risk factor. In COPD, respiratory microbiota is altered and airway inflammation is predominantly neutrophilic. OMVs from bacteria such as *Escherichia coli* and *Staphylococcus aureus* induce IL-17-mediated neutrophilic infiltration and alveolar damage, contributing to the development of emphysema^[64,65,69,71]^. Environmental exposure to dust-derived OMVs exerts comparable effects, supporting the hypothesis that chronic airway inflammation in COPD can be driven by microbial vesicles in the absence of overt infection^[64]^. Murine studies have demonstrated that repeated intranasal administration of *Escherichia coli* OMVs results in emphysema and airway inflammation through IL-17A-dependent pathways, with elevated levels of elastase^[71]^. Similarly, OMVs produced by *Pseudomonas aeruginosa*, a major pathogen in COPD and cystic fibrosis (CF), activate immune responses via TLR2 and TLR4, inducing chemokines such as Chemokine (C-X-C motif) ligand 1 (CXCL1) and Chemokine (C-C motif) ligand 2 (CCL2), pro-inflammatory cytokines including IL-1β, TNF-α, IL-6, and IFN-γ, and subsequent recruitment of neutrophils and macrophages^[70]^. The immune polarization elicited by mEVs appears to depend, in part, on the microbial lifestyle. Vesicles from extracellular Gram-negative bacteria typically promote Th17 responses and neutrophilic inflammation, whereas vesicles from intracellular bacteria like *Mycobacterium* induce Th1 responses dominated by IFN-γ production. This differential polarization is associated with distinct pathological outcomes: Th17-mediated inflammation may lead to airway hyperresponsiveness, fibrosis, and tissue remodeling, while Th1 responses are more closely linked to granulomatous inflammation and delayed-type hypersensitivity^[72]^. These immune pathways converge in their capacity to perpetuate chronic inflammation, destroy tissue architecture, and impair epithelial repair.

In CF, a common genetic disorder affecting the lungs and digestive system, caused by mutations in the CFTR gene, persistent infection with pathogens such as *Pseudomonas aeruginosa* and *Mycobacterium abscessus* contributes to the continuous production of mEVs. In this context, bacterial mEVs actively participate in biofilm initiation and maturation by delivering quorum-sensing molecules, enzymes, and small RNAs that enhance bacterial aggregation and surface adherence. These vesicles enhance biofilm formation, promote antibiotic resistance, and impair host antimicrobial defenses via transfer of bacterial small RNAs^[73,74]^. OMV-associated factors such as proteases, elastases, and LPS further exacerbate mucus hypersecretion and impair mucociliary clearance, reinforcing bacterial persistence in the CF lung. Experimental models have shown that bacterial OMVs can exacerbate lung remodeling, stimulate cytokine release, and disrupt clearance mechanisms, highlighting their role in maintaining chronic infection and structural lung damage. Evidence also links mEVs to fibrotic remodeling in the lung. Inhalation of dust-derived OMVs or exposure to OMVs from pathogenic bacteria may activate lung fibroblasts and drive epithelial-mesenchymal transition through IL-17-dependent signaling cascades^[75]^. These effects mirror pathological features found in advanced asthma and COPD and suggest that mEV-induced immune dysregulation may be a shared mechanism contributing to fibrosis across respiratory diseases.

Chronic exposure to mEVs may also contribute to lung cancer development. Prolonged mEV-driven inflammation promotes immune evasion by suppressing antigen presentation, inhibiting dendritic cell maturation, and favoring regulatory T cell expansion within the tumor microenvironment^[76]^. OMVs can modulate the tumor microenvironment, dampening cytotoxic immune responses and creating a permissive niche for tumor growth. Additionally, bacterial mEVs can carry immunomodulatory lipids, toxins, and nucleic acids that interfere with pattern recognition receptor signaling, thereby blunting anti-tumor immunity. Moreover, mEVs can be internalized by epithelial or neoplastic cells, potentially altering oncogenic signaling and enhancing drug resistance. Neutrophil-driven inflammation, initiated by mEVs, has also been associated with epithelial metaplasia and increased matrix metalloproteinase activity, processes implicated in malignant transformation. Elevated serum IgG levels against bacterial OMVs have been observed in patients with lung cancer, COPD, and non-eosinophilic asthma, suggesting that these vesicles might also serve as biomarkers for disease risk^[67]^. The environment represents an important source of mEVs. Indoor dust, particularly from mattresses and bedding, contains abundant EVs predominantly produced by potentially pathogenic bacteria, including *Pseudomonas, Acinetobacter, Enterobacter,* and *Staphylococcus*^[77,78]^. Repeated exposure to these particles has been shown to induce asthma-like inflammation and emphysema in murine models. Thus, inhaled mEVs represent a unique class of airborne pollutants capable of triggering chronic lung pathology in genetically predisposed individuals.

The airway microbiota, constantly shaped by inhalation, microaspiration, and mucociliary clearance, represents a dynamic source of vesicular exposure. Alterations in these regulatory mechanisms, combined with disruptions in gut-lung axis homeostasis, may enhance the pro-inflammatory potential of mEVs and sustain chronic immune activation.

**Figure 4:**
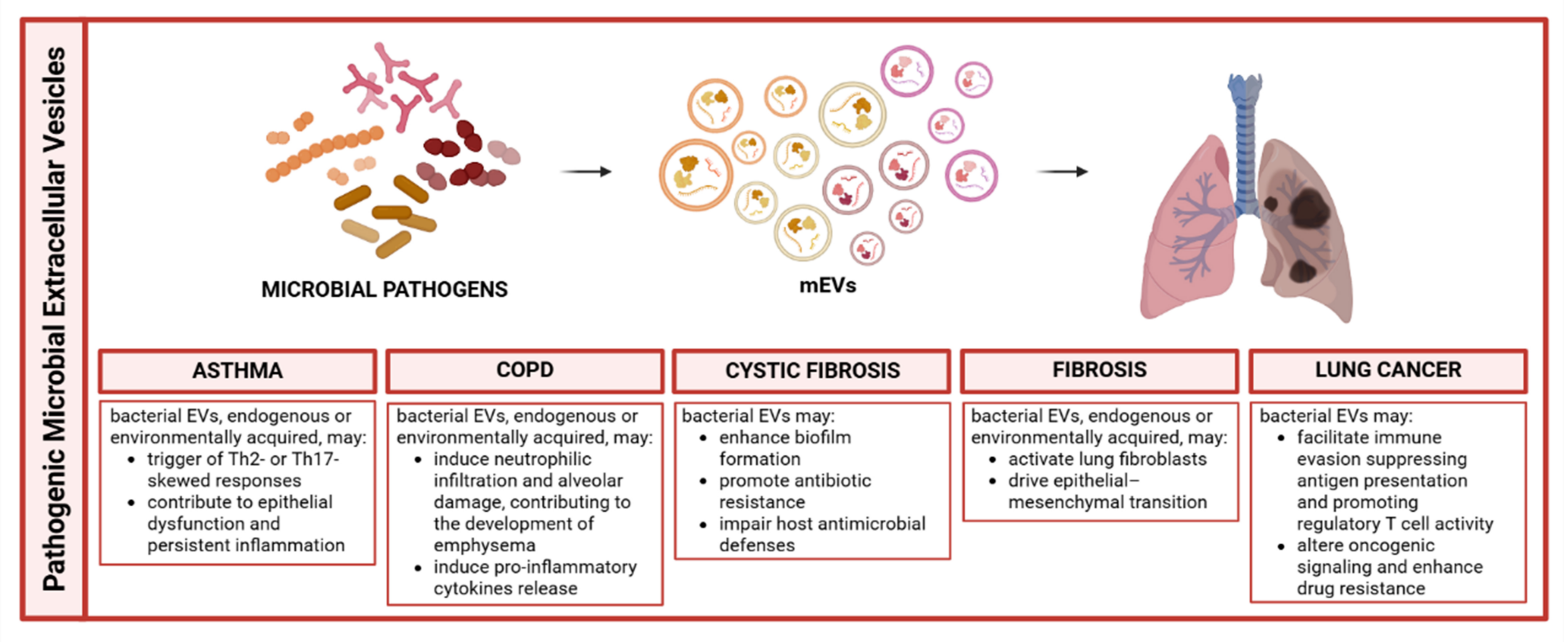
Schematic representation of pathogenic microbial-derived extracellular vesicles roles in pulmonary disease modulation. Created in BioRender. Bazzan, E. (2025) https://BioRender.com/036q7vv. mEVs: Microbial extracellular vesicles; EVs: extracellular vesicles; COPD: chronic obstructive pulmonary disease; Th2: T helper 2; Th17: T helper 17.

These insights into the role of mEVs in respiratory diseases (schematically summarized in Figure 4) have opened new diagnostic and therapeutic avenues. Metagenomic and proteomic analyses of vesicle cargo allow for disease-specific microbial profiling. Serum antibody responses against OMVs, for example, can help stratify asthma and COPD phenotypes and may predict clinical outcomes^[63,64]^. Additionally, mEVs are being investigated as delivery systems for immunomodulatory molecules, including siRNAs and therapeutic peptides, owing to their natural biocompatibility and ability to home to inflamed tissues. Preliminary studies suggest potential applications in modulating host responses to viral infections, including Severe Acute Respiratory Syndrome Coronavirus 2 (SARS-CoV-2)^[79,80]^.

## TRANSLATION CHALLENGES IN CLINICAL PERSPECTIVE

Despite growing interest in microbial EVs as key mediators of host-microbe interactions in the respiratory tract, their clinical translation remains challenging due to their dual functional nature. On one hand, pathogen-derived EVs have been implicated in the amplification of inflammation, immune dysregulation, and disease progression through the delivery of virulence factors, immunostimulatory molecules, and microbial nucleic acids. On the other hand, probiotic-derived EVs have emerged as potential immunomodulatory agents capable of promoting immune tolerance and tissue homeostasis. This functional dichotomy highlights the need for a nuanced and context-dependent evaluation of EV biological activity in clinical settings. Safety represents a central concern for both pathogenic and probiotic EV-based applications. While probiotic-derived EVs may offer advantages over live microorganisms, they can still carry microbial-associated molecular patterns such as LPS, LTA, or peptidoglycan fragments, which may trigger excessive or off-target immune responses in susceptible individuals. Conversely, pathogen-derived EVs pose inherent risks due to their enrichment in virulence-associated cargo, underscoring the importance of rigorous characterization, purification strategies, and safety profiling prior to any clinical exploitation. Additional challenges arise from the heterogeneity and context dependency of microbial EVs. EV composition, yield, and biological function are strongly influenced by microbial species, strain variability, environmental conditions, and host-microbe interactions. This variability complicates the identification of disease-specific or health-associated EV signatures and limits reproducibility across studies. Moreover, much of the current evidence is derived from *in vitro* or animal models, which may not fully capture the complexity of human respiratory diseases or probiotic-host interactions. Technical and regulatory barriers further limit clinical advancement. The lack of standardized methodologies for EV isolation, quantification, and functional assessment hampers cross-study comparisons and clinical validation. From a regulatory standpoint, microbial EVs do not currently fit well into existing frameworks, creating uncertainty regarding their classification as biologics, postbiotics, or therapeutic delivery systems. The absence of harmonized guidelines for quality control, potency testing, and long-term safety evaluation represents a major obstacle to their translation into clinical diagnostics or therapeutic interventions. Overall, overcoming these challenges will require standardized production and characterization pipelines, improved translational models, and a clearer regulatory landscape. A balanced understanding of both the pathogenic and probiotic properties of microbial EVs will be essential to safely harness their clinical potential while minimizing associated risks.

## FUTURE PERSPECTIVES

To fully harness the potential of these vesicles, future research must address the current gaps in mechanistic understanding and clinical implementation. Despite their growing relevance, our understanding of the molecular mechanisms underlying mEV-host interactions remains incomplete. A major challenge lies in the absence of robust *in vitro* and *in vivo* models that faithfully recapitulate human lung physiology and immune complexity. Future progress will depend on the development of physiologically relevant experimental models. Lung organoids are crucial for replicating the alveolar structure and mEV-epithelial interactions. Lung-on-chip systems are essential for studying mEV trafficking across the epithelial barrier under dynamic mechanical forces. Humanized animal models are necessary for assessing the systemic and long-term effects on adaptive immunity. Together, these models can more accurately reflect the complexity of mEV behavior in various respiratory disease settings. Simultaneously, well-designed clinical studies are essential to define the diagnostic, prognostic, and therapeutic value of microbial EVs in diverse patient populations. Translating these findings into clinical reality, however, is far from straightforward. We are currently facing a series of foundational and deeply intertwined hurdles that demand resolution before mEVs can reach the bedside. The most pressing concern is undoubtedly the safety profile. Specifically, the natural toxicity of Gram-negative OMVs, driven largely by LPS, remains a major barrier^[81,82]^. This makes the validation of detoxification methods, such as the deletion of the *msbB* gene, absolutely critical to reduce endotoxicity without completely abrogating biological activity^[83]^. Beyond safety, the industry is struggling with a lack of standardized Good Manufacturing Practice (GMP) protocols. Because these particles are so heterogeneous, scaling up production is a significant logistical challenge. We see significant batch-to-batch variability, and there is still no real consensus on what actually constitutes a “therapeutic unit”, is it the total protein content or the sheer number of particles or their functional potency? This ambiguity makes it incredibly difficult to move from successful lab experiments to predictable clinical outcomes^[84,16]^. Lastly, we have to navigate a murky regulatory landscape. mEVs currently sit in a complex gray area; they are often grouped together with Live Biotherapeutic Products (LBPs), yet their non-replicating nature clearly demands a more tailored regulatory framework that acknowledges their unique biological status^[44]^. Looking forward, several innovative directions are emerging. One promising avenue is the engineering of mEVs as targeted delivery vehicles for therapeutic molecules such as anti-inflammatory agents, RNA-based drugs, or genome-editing tools. Their natural biocompatibility, size, and ability to traverse biological barriers make them ideal candidates for precision pulmonary therapies. In parallel, the molecular cargo of airway mEVs, including proteins, lipids, and nucleic acids, offers exciting potential as non-invasive biomarkers for disease monitoring, patient stratification, and early detection.

Another emerging concept is the use of “designer probiotics”, engineered microbial strains optimized to secrete therapeutic EVs tailored to modulate host immunity or epithelial repair. These vesicles, as stable and non-replicating postbiotics, could circumvent the safety concerns associated with live microorganisms while retaining, or even enhancing, their beneficial effects. Integrating such strategies into personalized medicine frameworks could pave the way for novel interventions in chronic inflammatory diseases or even immuno-oncology.

In parallel to therapeutic applications, the molecular cargo of mEVs holds significant promise as non-invasive biomarkers for disease detection and monitoring in respiratory diseases^[85]^. Advanced AI-driven diagnostic platforms integrating machine learning algorithms such as gradient boosting machines, neural networks, and random forest models with microbial EV analysis have achieved exceptional diagnostic accuracies for asthma, COPD, and lung cancer^[86,87]^. Plasma and airway-derived microbial EVs have demonstrated diagnostic and prognostic value in pneumonia and other respiratory infections, enabling disease stratification and outcome prediction in mechanically ventilated patients^[88,65]^. However, clinical translation of mEV-based diagnostics requires addressing standardization of isolation protocols, integration with multi-omics platforms, and validation in large-scale clinical cohorts to fully realize their potential in precision respiratory medicine^[69,89]^.

Ultimately, mEVs challenge us to move beyond the traditional binary of pathogen *vs*. commensal. Instead, their biological impact is context-dependent, shaped by microbial origin, host status, and microenvironmental cues. Embracing this complexity, and investing in rigorous mechanistic and translational research, may allow us to harness mEVs not only as biomarkers or mechanistic clues but also as next-generation tools for the prevention, diagnosis, and treatment of respiratory diseases.

## CONCLUSIONS

mEVs represent a compelling paradox in respiratory medicine: they can act as both protective mediators and pathogenic triggers. This apparent duality is largely context-dependent: the vesicle dose, cargo composition, route of exposure, and the immune status of the host collectively determine whether an mEV promotes tolerance or triggers inflammation (Table 1 explained this possible dual role). Systematic dose-response studies and standardized cargo profiling are therefore essential to predict biological outcomes and to guide the safe development of therapeutic applications. On one hand, mEVs derived from commensal or probiotic bacteria are emerging as promising immunomodulatory agents capable of restoring immune balance, reinforcing epithelial integrity, and promoting respiratory homeostasis. On the other hand, mEVs released by pathogenic or environmental microorganisms contribute to airway inflammation, epithelial dysfunction, and the progression of chronic respiratory diseases including asthma, COPD, and lung cancer. This dualistic nature underscores the urgent need to shift from viewing mEVs merely as microbial by-products to recognizing them as dynamic biological effectors with both harmful and therapeutic potential in the lung microenvironment.

**Table 1:** Comparative table summarizing the most important features of key studies comparing probiotic and pathogenic EVs, including EV source, experimental model, EV cargo, and main findings or outcomes

**Study**	**EV source**	**Model**	**Key findings/outcome**	**Cargo**
Liu *et al*., 2024^[53]^	*Clostridium butyricum* EVs	LPS-induced ALI (mouse)	Reduced IL-6/TNF-α in serum, BALF and lung tissues; increased ZO-1/occludin; improved histology	EV protein analysis described; results not reported
Zhao *et al*., 2025^[54]^	*Lactiplantibacillus plantarum*- derived EVs	LPS-induced ALI (mouse, mouse macrophage cell line)	Protected against macrophage polarization and ferroptosis	EV protein analysis described; results not reported
Lee *et al*., 2022^[57]^	*Lactococcus lactis*- derived EVs	Allergic asthmatic mice	Decreased IL-5/IL-13; increased IFN-γ in the BALF	The most abundant proteins were pyruvate kinase, arginine deaminase, ornithine transcarbamylase
Sim *et al*., 2023^[56]^	*Micrococcus luteus* EVs	Asthma (mouse, A549 cells)	Decreased neutrophils; reduced IL-1β/IL-17 in the BALF; lowered IL-8 in A549	Not specified
Schmid *et al*., 2023^[61]^	*Escherichia coli* OMVs	Ovalbumin- induced allergic airway model (mouse, splenocytes and bone marrow-derived dendritic cells)	Reduced airway hyperresponsiveness; eosinophilia, decreased Th2 cytokines and mucus production	136 proteins (including flagellar proteins FlgL1, FlgL3, FlgE, FlgG, FliC) were detected exclusively in the vesicles
Preet *et al*., 2025^[62]^	Bifidobacterium- derived EVs	Human (A549, H460) and murine (LL/2) lung cancer cells Syngeneic mouse model	Modulated PD-L1 expression and key cytokines (IFN-γ, IL-2); synergized anti-tumor effect of anti-PD-1; increase in tumor-infiltrating CD8+ T-cells	Not specified
Keshavarz Aziziraftar *et al*., 2024^[40]^	*A. muciniphila*- EVs	CCl4-induced pulmonary fibrosis (mouse)	Downregulated fibrosis markers *α-SMA, Col1, PDGF*, and *TIMP;* overexpression of TNF-α, IL-1β and TGF-β; elevated production of anti-inflammatory cytokine IL-10	Not specified
Park *et al*., 2013^[63]^	*P. aeruginosa* OMVs	Wild-type and TLR2/TLR4 knockout mice; MH-S cell lines, human embryonic kidney cell line	Activated TLR2/4 signaling; induced chemokines and pro-inflammatory cytokines; immune cell recruitment	Not specified
Kim *et al*., 2015^[71]^	*E.coli* EVs and indoor dust-EVs	IL-17A-deficient mice; TLR2/4 -deficient mice; MH-S cells	Emphysema and airway inflammation through IL-17A-dependent pathways; elevated levels of elastase	Not specified
Kim *et al*., 2013^[72]^	1.Extracellular Gram - bacteria EVs 2. Intracellular bacteria EVs	C57BL/6 mice; macrophages, ATCC, BEAS-2B cells; patients with atopic asthma, rhinitis or dermatitis	1. Promoted Th17 responses and neutrophilic inflammation 2. Induced Th1 responses dominated by IFN-γ production	Not specified
Yang *et al*., 2019^[75]^	Bacterial OMVs from *B.ovatus, B.stercoris, P.melaninogenica*	Mouse lungs Macrophages	Induced IL-17A/B expression, promoted Th17 differentiation, and fibrosis	Not specified

EVs: Extracellular vesicles; OMVs: outer membrane vesicles; LPS: lipopolysaccharide; ALI: acute lung injury; BALF: bronchoalveolar lavage fluid; IL: interleukin; TNF-α: tumor necrosis factor alpha; ZO-1: zonula occludens-1; IFN-γ: interferon gamma; Th1: T helper 1; Th2: T helper 2; Th17: T helper 17; A549: human lung adenocarcinoma cell line; H460: human lung cancer cell line; LL/2: Lewis lung carcinoma cell line; PD-L1: programmed death-ligand 1; PD-1: programmed cell death protein 1; CD8+: cluster of differentiation 8 positive T cells; α-SMA: alpha-smooth muscle actin; Col1: collagen type I; PDGF: platelet-derived growth factor; TIMP: tissue inhibitor of metalloproteinases; TGF-β: transforming growth factor beta; CCl4: carbon tetrachloride; TLR: Toll-like receptor; MH-S: murine alveolar macrophage cell line; ATCC: American Type Culture Collection; BEAS-2B: human bronchial epithelial cell line; IL-17A: interleukin-17A; IL-17B: interleukin-17B.

## DECLARATIONS

### AI and AI-assisted tools statement

During the preparation of this manuscript, the AI tool DeepL (DeepL SE, latest available version at the time of use) was used solely for language editing. The exact version number and release date are not publicly specified by the provider. The tool did not influence the study design, data collection, analysis, interpretation, or the scientific content of the work. All authors take full responsibility for the accuracy, integrity, and final content of the manuscript.

### Copyright

© The Author(s) 2026.
